# Host preference of the chinch bug, Blissus occiduus

**DOI:** 10.1673/1536-2442(2006)6[1:HPOTCB]2.0.CO;2

**Published:** 2006-06-08

**Authors:** Thomas E. Eickhoff, Frederick P. Baxendale, Tiffany M. Heng-Moss

**Affiliations:** Department of Entomology, University of Nebraska, Lincoln, NE 68583

**Keywords:** western chinch bug, buffalograss, zoysiagrass, plant resistance

## Abstract

The chinch bug, Blissus occiduus Barber (Hemiptera: Blissidae), is an important pest of buffalograss, Buchloë dactyloides (Nutall) Engelmann and potentially other turfgrass, crop, and non-crop hosts. Choice studies documented the number of B. occiduus present on selected turfgrasses, crops and weeds, and provided important insights into the host preferences of this chinch bug. Grasses with the most chinch bugs present included the warm-season turfgrasses B. dactyloides, zoysiagrass, Zoysia japonica Steudel, bermudagrass, Cynodon dactylon (L.) Pers., and St. Augustinegrass, Stenotaphrum secundatum (Walt.) Kuntze. The other grasses tested, green foxtail, Setaria viridis (L.) Beauv, Kentucky bluegrass, Poa pratensis L., perennial ryegrass, Lolium perenne L., rye, Secale cereale L., sorghum, Sorghum bicolor (L.) Moench, tall fescue, Festuca arundinacea Schreb. and wheat Tritium aestivum L. had significantly fewer chinch bugs. Buffalograss and zoysiagrass had the highest numbers of chinch bugs among the warm-season grasses and the buffalograss cultivars ‘86–120’ and ‘PX-3-5-1’ had more chinch bugs than the zoysiagrass cultivars ‘Meyers’ and ‘El Toro’ after the two hour evaluation time.

## Introduction

Buffalograss, Buchloë dactyloides (Nutall) Engelmann, is a warm-season turfgrass that is drought tolerant and has low maintenance requirements, offering an alternative to more traditional high input turfgrasses typically used in turf settings. In the early 1990's, the chinch bug, Blissus occiduus Barber emerged as a serious pest of buffalograss grown for turf (Baxendale et al. 1999). During the summer of 2000, B. occiduus was discovered for the first time causing severe injury to stands of zoysiagrass, Zoysia japonica Steudel, in southeast Nebraska (Eickhoff 2002), increasing the importance of this chinch bug as a serious pest of turfgrass. First described by Barber (1918), the reported distribution of B. occiduus currently includes California, Colorado, Kansas, Montana, Nebraska, and New Mexico in the United States, and Alberta, British Columbia, Manitoba, and Saskatchewan in Canada (Bird and Mitchener 1950; Slater 1964; Baxendale et al. 1999). Reported hosts of B. occiduus include corn, Zea mays L. (Ferris 1920), sugarcane, Saccharum officinarum L. (Ferris 1920; Box 1953; Slater 1976) wheat, Triticum aestivum L. (Bird and Mitchner 1950), barley, Hordeum spp., brome, Bromus spp. (Farstad and Staff 1951) and buffalograss (Baxendale et al. 1999). In addition to the previously mentioned hosts, greenhouse studies by Eickhoff et al. (2004) identified several additional potential grass hosts of B. occiduus including: bermudagrass, Cynodon dactylon (L.), green foxtail, Setaria viridis (L.) Beauv., Kentucky bluegrass, Poa pratensis L., large crabgrass, Digitaria sanguinalis (L.) Scop., perennial ryegrass, Lolium perenne L., rye, Secale cereale L., sorghum, Sorghum bicolor (L.) and yellow foxtail, Setaria glauca. The ability of this chinch bug to feed and reproduce on a wide variety of the grasses present in both agronomic and horticultural settings emphasizes the need for further information regarding the host preference of B. occiduus in habitats containing several suitable hosts.

It has been well documented that the common chinch bug, Blissus leucopterus leucopterus Say, can be a serious pest of corn, sorghum, millet, rye, many bunch-grasses species, and both cool and warm season turfgrasses (Brandenburg et al. 1995). B. l. leucopterus often migrates in large numbers from maturing grain fields to alternate hosts, including other crop fields and turf. Interestingly, Slater (1976) noted that B. l. leucopterus rarely inhabits the most abundant grass species in its environment. Instead, it often occurs on inconspicuous grasses while being completely absent from related grass species occurring in the same area. Certain Blissus species are known to move from reproductive hosts to a secondary food supply when the original host becomes unsuitable or is no longer available. These chinch bugs may be capable of reproducing on the ‘secondary host’, but only do so in the absence of their preferred host (Slater 1976). This behavior has important implications because alternate hosts often permit chinch bugs to reach high numbers while going relatively undetected.

Although B. occiduus has not been previously reported to move among multiple hosts over the course of its development, it remains unclear if this chinch bug strictly prefers buffalograss or will move to secondary hosts adjacent to buffalograss. The emergence of B. occiduus as a potential pest of zoysiagrass warrants further investigation on its host preference. Greenhouse studies have documented that B. occiduus will feed and cause serious damage on a number of important turfgrass, crop and weed species (Eickhoff et al. 2004). Furthermore, these studies revealed that B. occiduus can reproduce on many of the same hosts in which it causes serious damage. Since several of the grasses (green foxtail, wheat and Kentucky bluegrass) identified as potential hosts of B. occiduus are commonly found in or adjacent to horticultural and agronomic cropping systems, it is important to determine if any are preferred by B. occiduus. Knowledge of the preferred hosts of B. occiduus will assist in monitoring B. occiduus populations by allowing earlier detection of chinch bug infestations before they build to damaging levels. Further, increased knowledge of B. occiduus biology and chinch bug-host interactions will aid in the development and/or more efficient use of improved management approaches including biological control, plant resistance, habitat modification, proper maintenance practices and chemical controls. The objective of this study was to investigate the alternant host preferences of B. occiduus in the presence of its preferred host buffalograss.

## Materials and Methods

Two choice studies were conducted using growth chamber conditions to determine B. occiduus host preference for selected turfgrass, crop and weed species. The following eleven grasses were evaluated in each of the studies: bermudagrass, cultivar unknown; buffalograss, NE 86-120; Kentucky bluegrass, ‘Eclipse’; perennial ryegrass, (nonendophyte-enhanced), “Saturn II”; St. Augustinegrass, “Raleigh”; tall fescue, Festuca arundinacea Schreb., “Falcon II.”; zoysiagrass, ‘El Toro’; rye, cultivar unknown; sorghum, “Garst 5715 ” and wheat, “Hondo” and the agronomic weed species green foxtail. These grasses were selected based on their susceptibility to B. occiduus as reported by Eickhoff et al. (2004).

Following methods outlined by Smith et al. (1994), all chinch bugs used were collected from buffalograss research plots at the University of Nebraska's John Seaton Anderson (JSA) Research Facility near Mead, Nebraska by vacuuming the soil surface with a modified ECHO Shred ‘N Vac (Model #2400, ECHO Incorporated). Chinch bugs were preconditioned by starving them for 24 h prior to the initiation of the experiment while being held under laboratory conditions (26 ± 3° C 16:8 L:D photoperiod).

**Figure 1.**
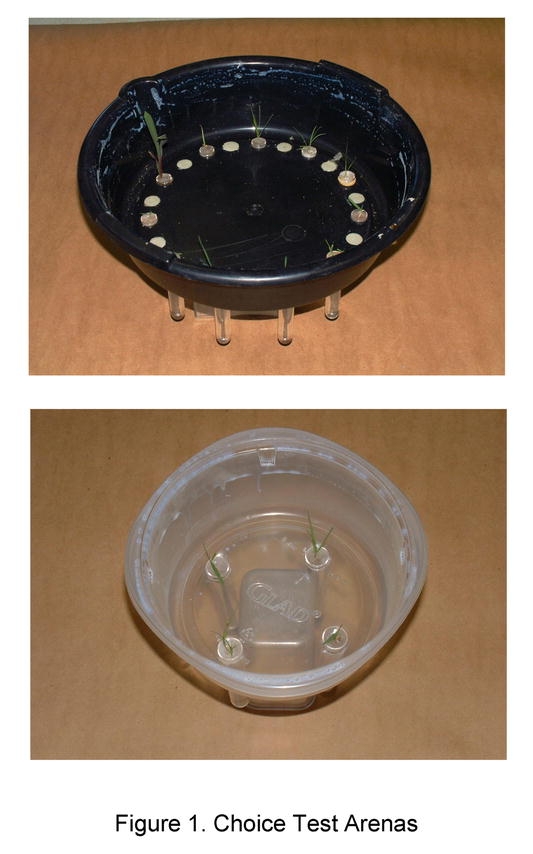
Choice Test Arenas

The buffalograss selection NE86-120 was used as the susceptible check in all experiments because it is highly preferred by B. occiduus and shows only moderate resistance (tolerance) to chinch bug feeding (Heng-Moss et al. 2003). Sod plugs of NE86-120, 10.6 cm diameter by 8 cm deep, were extracted from research plots at the JSA Research Facility, and served as the vegetative buffalograss source for these studies. The remaining warm season grasses (St. Augustinegrass, bermudagrass and zoysiagrass) were acquired from Turfgrass America in Granville, Texas. These grasses were established in the greenhouse in 35.56 cm by 50.8 cm flats. All warm season turfgrasses were vegetatively propagated by planting individual stolons or rhizomes of each grass in ‘SC-10 Super Cell’ Single Cell Cone-tainers® (3.8 cm diameter by 21 cm depth) (Stuewe & Sons, Inc.) containing a potting mixture of sand-soil-peat-perlite in a 0.66:0.33:1:1 ratio three weeks prior to initiation of experiments. The warm-season turfgrasses were trimmed to the soil surface one week before initiation of the experiment. The remaining grasses were grown from seed in Cone-tainers as previously described. The faster germinating grasses (green foxtail, rye and wheat) and the slow germinating grasses (Kentucky bluegrass, perennial ryegrass, sorghum and tall fescue) were planted 4 and 7 days prior to the initiation of the experiment, respectively. Foam plugs (approximately 1.8 cm in diameter) were slit vertically and individual plants inserted. The plug was then placed in a vial (1.7 cm diameter by 9 cm deep) of water and sealed with Parafilm. Vials with grasses were randomly inserted into 1.7 cm diameter holes drilled in circular test arenas (33.5 cm diameter by 8.5 cm deep) (Fig. 1). A 2 cm band of petroleum jelly was applied to the top portion of each arena to prevent chinch bug escape.

Fifty 4^th^ and 5^th^ instar chinch bugs (sex unknown) were released in the center of each arena. The number of chinch bugs on each grass was visually determined at 1, 2, 4, 8, 24, 48, and 72 h after chinch bug introduction. Based on the results of these studies a third choice study was conducted using four highly preferred buffalograss and zoysiagrass cultivars (Eickhoff unpublished data). The grasses evaluated in this study included the buffalograsses NE86-120 and ‘PX-3-5-1’, and the zoysiagrass cultivars ‘Myers’ and ‘El Toro’. Turfgrasses used in this study were acquired from the same sources and established in the greenhouse as previously described. Chinch bugs were again collected from buffalograss research plots.

Individual plants were placed in vials of water and sealed with paraffin wax heated to 63 ± 2° C. Vials with grasses were randomly inserted into 1.7 cm diameter holes drilled in circular test arenas (16 cm diameter by 8 cm deep).

Twenty-five 4^th^ and 5^th^ instar chinch bugs (sex unknown) were released in the center of each arena. The number of chinch bugs used in this study was reduced from the previous studies since smaller test arenas and fewer grasses were used. The number of chinch bugs on each grass was visually determined at 1, 2, 4, 8, 24, 48, and 72 h after chinch bug introduction.

All experiments were conducted in a growth chamber that was maintained at 28 ± 2° C under 24 h lighting. The experimental design was a randomized complete block design with ten replications for choice studies 1 and 2, and fifteen replications for choice study 3.

### Statistical Analyses

Mixed model analyses (PROC MIXED, SAS Institute 1999) were conducted to identify differences in chinch bug preference among the evaluated grasses. The residuals from the mixed model analyses were inspected to check the model assumptions of normality and constant variance. No significant violations of these assumptions were discovered and when appropriate, means were separated using Fisher's LSD procedure.

## Results

### Choice studies 1 and 2

No significant differences (*P* > 0.05) between choice studies 1 and 2 were detected, therefore the data were pooled. A significant interaction (F = 2.29; df = 60, 1254; *P* < 0.0001) between grass species and evaluation time was detected. This interaction likely reflects changes in chinch bug numbers on the different grasses over time as well as significant differences between grasses at the individual evaluation times. When chinch bugs were first introduced, for the first 10–15 minutes the insects appeared to move randomly to a grass host. If the first grass visited was not a preferred host, the chinch bugs gradually moved to more preferred hosts over the next 72 h (Table 1).

**Table 1.**
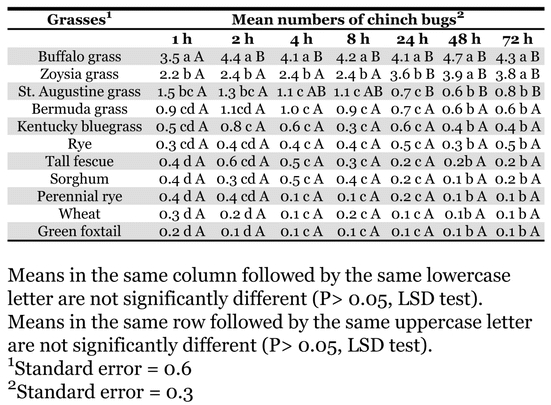
Blissus occiduus numbers on selected grasses over time.

Buffalograss had significantly more chinch bugs present than any other grass, at all evaluation times except for zoysiagrass at 48 and 72 h after introduction (Table 1). Zoysiagrass had significantly more chinch bugs present than all the remaining grasses, at all evaluation times except for St. Augustinegrass at 1 and 2 h after introduction. This suggests that buffalograss and zoysiagrass are highly preferred by B. occiduus over the other grasses tested. In addition, these results indicate that even though many grasses are susceptible to B. occiduus feeding injury, they are not highly preferred. This research also suggests that B. occiduus may be more likely to colonize buffalograss and/or zoysiagrass before other grasses. Interestingly, the warm season turfgrasses (bermudagrass, buffalograss, St. Augustinegrass and zoysiagrass) were, in general, more preferred than any of the cool-season turfgrasses and other grasses evaluated. During the course of this study, more than 50% of the chinch bugs selected either buffalograss or zoysiagrass in the first 8 hours, and by 24 h this number rose to over 70%. Collectively, the warm season turfgrasses made up over 75% of the grasses chosen in the first 4 h. This rose to over 80% during the remaining evaluation times. Combined, the remaining grasses never exceeded 25%. This number decreased over the remaining evaluations to less than 10% at 48 h. Further research is needed to determine which grasses B. occiduus would select in the absence of buffalograss and zoysiagrass.

### Choice study 3

Significant differences in chinch bug preference were observed among the four warm-season turfgrasses evaluated (F = 5.68; df = 3, 392; *P* < 0.0008) and seven evaluation times (F = 5.47; df = 6, 392; *P* < 0.0001) (Table 2). The greatest differences were detected at 24, 48, and 72 h after chinch bug introduction. The buffalograss NE86-120 was the most preferred selection of all grasses tested beginning at 8 h after introduction, which supports the findings of Heng-Moss et al. (2003). Numerically PX-3-5-1 was more preferred by B. occiduus when compared to the zoysiagrasses ‘El Toro’ and ‘Myers’. No statistical differences in chinch bug numbers were detected between the two zoysiagrass cultivars tested. This study establishes B. occiduus' preference for buffalograss over zoysiagrass.

**Table 2.**
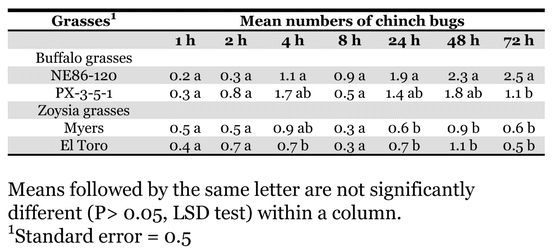
Blissus occiduus numbers on buffalograss and zoysiagrass over time.

## Discussion

These studies suggest the preference of B. occiduus for warm-season turfgrasses over all other turfgrass, crop and weed species tested as well as document the host preferences of B. occiduus among these grass hosts. Further, B. occiduus has a high preference for buffalograss and zoysiagrass when compared to other warm-season turfgrasses.

This research provides evidence that B. occiduus may initially select a host based on morphological characteristics and/or plant architecture. Lynch (1987) suggested that goosegrass and ‘Tifton 78’ bermudagrass were highly preferred by nymphal B. l. leucopterus over all other grasses tested because of differences in grass blade width. In this study, the warm season turfgrasses (buffalograss, zoysiagrass, St. Augustinegrass and bermudagrass) were initially selected over all of the other evaluated grasses (turfgrass, crop and weed). These stoloniferous turfgrasses may provide more cover for host-seeking B. occiduus than the non-stoloniferous species. Interestingly, two of these warm season grasses (St. Augustinegrass and bermudagrass) were neither highly preferred feeding or reproductive hosts (Eickhoff et al. 2004). It is possible that B. occiduus initially selected these grasses because of the potential feeding sites associated with their stoloniferous growth habit. This hypothesis is supported by the observed decrease in B. occiduus numbers on St. Augustinegrass over time.

Blissus occiduus preference for buffalograss and zoysiagrass has important management implications for this pest. Buffalograss and zoysiagrass stands in close proximity may be at higher risk of chinch bug infestations than when adjacent to other turfgrass species. Further, the selection of highly preferred cultivars/selections such as the buffalograsses NE86-120 and ‘PX-3-5-1’, and the zoysiagrasses ‘Myers’ and ‘El Toro’ should be avoided or closely monitored to ensure early detection and effective management of chinch bug infestations. In regions where warm-season turfgrasses are the dominate species, selection of less preferred turfgrass species (i.e. St. Augustinegrass ) maybe an effective strategy for avoiding B. occiduus infestations. However, B. occiduus' potential to cause damage and reproduce on a wide variety of grass hosts must be considered, and the potential infestation of these alternative grasses should not be discounted especially where large areas of preferred hosts have become severely damaged by heavy infestations of B. occiduus.
